# A Systematic Review of Gene Expression Studies in Critically Ill Patients with Sepsis and Community-Acquired Pneumonia

**DOI:** 10.3390/biomedicines11102755

**Published:** 2023-10-11

**Authors:** Diego Viasus, Lara Nonell, Carlos Restrepo, Fabian Figueroa, Carla Donado-Mazarrón, Jordi Carratalà

**Affiliations:** 1 Department of Medicine, Division of Health Sciences, Universidad del Norte and Hospital Universidad del Norte, Barranquilla 081001, Colombia; 2 Departament de Biociències, Universitat de Vic—Universitat Central de Catalunya, 08500 Barcelona, Spain; laranonell@gmail.com; 3 Department of Infectious Diseases, Bellvitge University Hospital, Bellvitge Biomedical Research Institute (IDIBELL), University of Barcelona, 08907 Barcelona, Spain; carladonado98@gmail.com; 4 Centro de Investigación Biomédica en Red de Enfermedades Infecciosas (CIBERINFEC), Instituto de Salud Carlos III, 28029 Madrid, Spain

**Keywords:** community-acquired pneumonia, gene expression profile, gene set enrichment analysis, Reactome

## Abstract

(1) Background: Sepsis is present in nearly 90% of critically ill patients with community-acquired pneumonia (CAP). This systematic review updates the information on studies that have assessed gene expression profiles in critically ill septic patients with CAP. (2) Methods: We searched for studies that satisfied the following criteria: (a) expression profile in critically ill patients with sepsis due to CAP, (b) presence of a control group, and (c) adult patients. Over-representation analysis was performed with clusterProfiler using the Hallmark and Reactome collections. (3) Results: A total of 4312 differentially expressed genes (DEGs) and sRNAs were included in the enrichment analysis. In the Hallmark collection, genes regulated by nuclear factor kappa B in response to tumor necrosis factor, genes upregulated by signal transducer and activator of transcription 5 in response to interleukin 2 stimulation, genes upregulated in response to interferon-gamma, genes defining the inflammatory response, a subgroup of genes regulated by MYC—version 1 (v1), and genes upregulated during transplant rejection were significantly enriched in critically ill septic patients with CAP. Moreover, 88 pathways were identified in the Reactome database. (4) Conclusions: This study summarizes the reported DEGs in critically ill septic patients with CAP and investigates their functional implications. The results highlight the complexity of immune responses during CAP.

## 1. Introduction

Community-acquired pneumonia (CAP) remains one of the most common infectious diseases and a major cause of mortality worldwide [1]. CAP is defined as an acute infection of the lungs (alveoli and distal airways) acquired outside hospitals or long-term care facilities. Typical clinical manifestations included fever, cough, mucopurulent sputum, pleuritic chest pain, and dyspnea [2,3]. Acute respiratory distress syndrome, sepsis, pleural effusion, and worsened preexisting comorbidities are complications of CAP. Viruses and bacteria are the most frequent etiology. Studies have found that influenza is the most common cause of viral CAP, and *Streptococcus pneumoniae*, *Klebsiella pneumoniae*, *Haemophilus influenzae*, anaerobes, and atypical bacteria (i.e., *Mycoplasma pneumoniae* and *Legionella species*) represent common causes of bacterial CAP [2]. A considerable number of factors related to sociodemographic characteristics, clinical manifestations or complications, and causative pathogens can affect mortality in CAP patients [4]. Similarly, studies have shown that an inappropriate inflammatory response is a major cause of treatment failure and mortality in patients with CAP [5,6,7]. Kellum et al. [7] found that high levels of both proinflammatory and anti-inflammatory cytokines were related to higher risk of mortality. The overall mortality rate of CAP has changed little in recent decades, and in patients admitted to intensive care units (ICU), it continues to exceed 20% [8,9].

Moreover, sepsis is a medical emergency characterized by a dysregulated host inflammatory and immune response to an infectious process that results in organ injury and death [10]. Studies have documented that the incidence of sepsis has increased continuously in recent decades and can be attributed to different factors, such as the aging of the population, resistance to antibiotics, the greater number of immunosuppressed patients, and the increased need for invasive procedures [10,11]. Despite an increase in knowledge of the pathophysiological characteristics of sepsis, the ability to intervene and modify the evolution of the disease has not been entirely satisfactory. Patients who develop sepsis during an infectious process have a high risk of mortality and represent about 20% of all global deaths [10,12].

Sepsis is common in patients with CAP; it is present in more than 50% of those admitted to the ward and in nearly 90% of patients treated in the ICU [13]. Sepsis and organ dysfunction in patients with CAP are risk factors for poor outcomes, especially in patients who present with septic shock or require mechanical ventilation [9,13].

Timely initiation of appropriate broad-spectrum antibiotic therapy is the cornerstone of treatment for CAP and sepsis. Current guidelines recommend a β-lactam plus a macrolide or fluoroquinolone monotherapy as empiric antibiotic therapy for hospitalized patients with CAP. Other recommendations to take into account in the management of CAP and sepsis include the appropriate use of fluids, intensive support of organ dysfunction, control of the infectious focus, nutritional support, glucose management, serial analytical monitoring, and corticosteroids [3,14]. In recent years, new therapeutic interventions for CAP and sepsis have been evaluated. In this regard, studies have documented that mesenchymal stem cells have immunomodulatory properties in various diseases. These cells also been described as having angiogenic, antiapoptotic, antibacterial and tissue repair activity [15,16]. The above characteristics have made mesenchymal stem cells candidates for the treatment of CAP and sepsis [15]. A meta-analysis of preclinical studies found that mesenchymal stem cell therapy was associated with reduced mortality in animal models of sepsis [16]. Recently, several clinical phase 1/2 trials have evaluated mesenchymal stem cell treatment in critically ill patients with acute respiratory distress syndrome (ARDS) and sepsis. These studies found that mesenchymal stem cell therapy is safe, although the number of patients who have received therapy to date is small [17].

Information derived from gene expression studies may improve our understanding of the immune response during sepsis and CAP and may help to identify new pathways or candidate biomarkers and targets for possible interventions. Recently, interesting studies on the blood transcriptome of patients with sepsis and CAP have been published [18,19,20]. Gene expression profiling in septic patients has revealed differential regulation compared to healthy volunteers or non-septic patients with CAP. Genes related to host defenses and inflammatory responses have been found to be upregulated during sepsis. Furthermore, gene expression studies have allowed the stratification of CAP as a heterogeneous disease into subtypes with possible diagnostic and/or prognostic impacts [21].

This systematic review updates the information on studies that have evaluated the expression profiles in critically ill septic patients with CAP, focusing on identifying which genes are differentially expressed in these patients compared to controls (i.e., critically ill adult patients without sepsis, healthy adults, or others).

## 2. Materials and Methods

This systematic review was conducted in accordance with the Preferred Reporting Items for Systematic Reviews and Meta-Analyses (PRISMA) guidelines [22]. We searched the PubMed/MEDLINE, Cochrane Central Register of Controlled Trials (CENTRAL), and LILACS (Literatura Latino-Americana e do Caribe de Informação em Ciências da Saúde) databases from inception to December 2021 for studies that satisfied the following eligibility criteria: (a) expression profile in critically ill patients with sepsis due to CAP, (b) presence of a control group, and (c) adult age. Our search also identified relevant publications from the references of the articles included. The search terms used were “community-acquired pneumonia”, “sepsis”, “intensive care unit”, “gene expression”, and “transcriptome”. We included observational studies (cohort and case-control studies) in English and Spanish, excluding studies on animals and pediatric populations, abstracts, and case reports (<5 cases). Likewise, patients with SARS-CoV-2 disease (COVID-19) were also excluded. The study was registered with the International Registry of Systematic Reviews/Meta-Analyses (PROSPERO CRD42022342685).

Two pairs of reviewers (DV, CR, FF, and CD) assessed titles and abstracts for inclusion. Eligibility of these article was determined by independent review of the full text. If any disagreement occurred, it was resolved by another reviewer (JC). The following information was extracted from the included articles and collected on a standardized form created for the review: author, year, journal, country in which the study was conducted, original study design, sample size, sample size of the subgroup, tissue investigated, methods for analyzing gene expression changes, and differentially expressed genes comparing patients with sepsis due to CAP and a comparator group. A modified Quality of Genetic Association Studies (Q-Genie) tool was used for critical appraisal of the included studies [23]. Two reviewers (CR and DV) independently performed these quality assessments. If any disagreement occurred, it was resolved by consensus between the reviewers.

### Statistical Analysis

A description was made on the basis of the data extracted and main findings of each study. Datasets were assessed for differentially expressed genes (DEGs) and sRNAs between critically ill patients with sepsis due to CAP and controls. All DEGs were filtered to have an absolute fold change (FC) > 1.7 and *p* value < 0.05, based on the maximum FC threshold reported by Severino et al. [24]. Over-representation analysis (ORA) was performed with clusterProfiler [25] using Hallmark and Reactome (C2.Reactome) collections from the MSigDB (v. 2023). Gene sets from a collection were considered significative if adj.p.val < 0.05. Analyses were performed in R [26]. Table S1 shows the codes used in R to analyze the data.

## 3. Results

**Figure 1:**
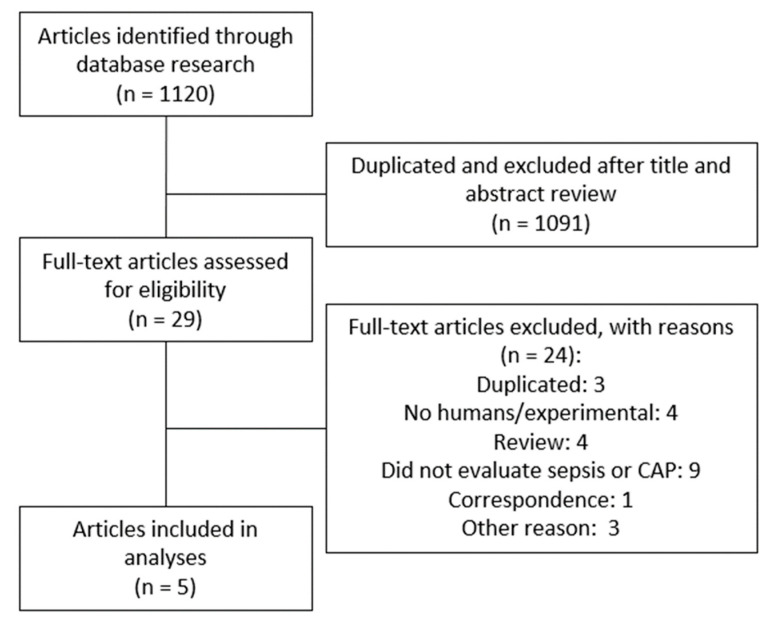
Flow diagram of the study selection process.

Figure 1 shows the PRISMA flowchart describing the process of the systematic literature search to identify all eligible studies. We identified and screened 1120 papers. A total of 1091 articles were excluded because they did not meet the inclusion criteria based on title and abstract or duplication. Therefore, 29 articles were considered relevant for full-text review and eligibility, of which five were included in this systematic review [24,27,28,29,30].

The main characteristics of the studies included are summarized in Table 1. In all, there were 231 critically ill patients with sepsis due to CAP and 71 healthy controls. Studies investigated expression in whole or peripheral blood leukocytes and monocytes. Three of the studies used gene expression microarrays, one using next-generation RNA sequencing (RNA-Seq) studied small RNA, and the other estimated the expression of 35 genes involved in NLR inflammasome pathways using real-time polymerase chain reaction (RT-PCR). The quality assessment scores of the included studies are found in Table S2. Total quality assessment scores ranged between 52 and 56 (median = 53).

**Figure 2:**
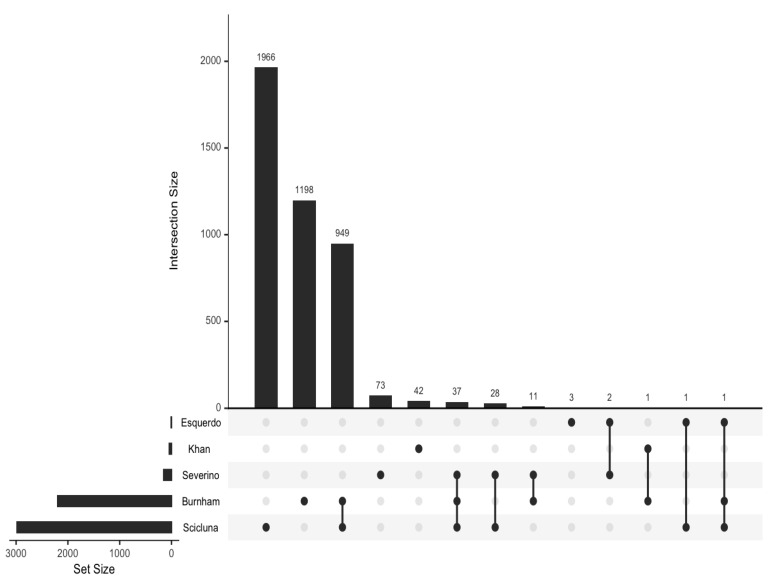
UpSet plot to visualize the overlap between sets of differentially expressed genes.

**Figure 3:**
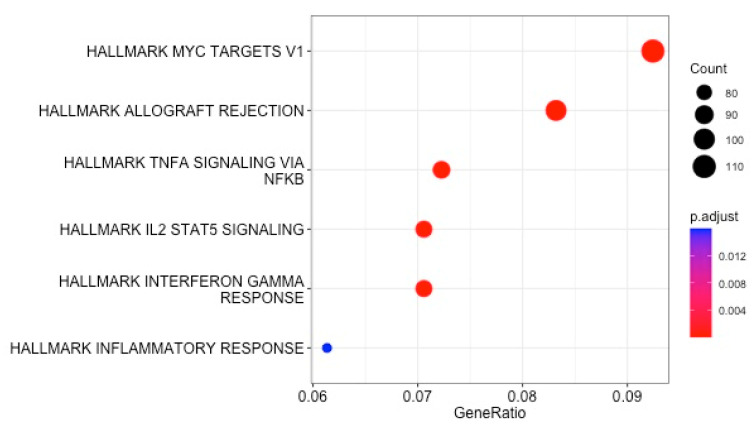
Enrichment analysis using the Hallmark collection from the MSigDB: dot plot of enriched gene sets.

All 4312 reported DEGs and small RNAs (FC > 1.7 and *p* value < 0.05) (Table S3) were included in an enrichment analysis using the Hallmark and Reactome gene set collections from the Molecular Signatures Database (mSigDB) (v. 2023). Minimal overlap of the DEGs from the included studies was noted (Figure 2). Regarding the Hallmark gene set collection, the following DEGs were significantly over-represented in critically ill septic patients with CAP: genes regulated by nuclear factor kappa B (NF-kB) in response to tumor necrosis factor (TNF), genes upregulated by signal transducer and activator of transcription 5 (STAT5) in response to interleukin 2 (IL2) stimulation, genes upregulated in response to interferon-gamma (IFN-γ), genes defining an inflammatory response, a subgroup of genes regulated by MYC—version 1 (v1), and genes upregulated during transplant rejection (Table 2 and Figure 3).

**Figure 4:**
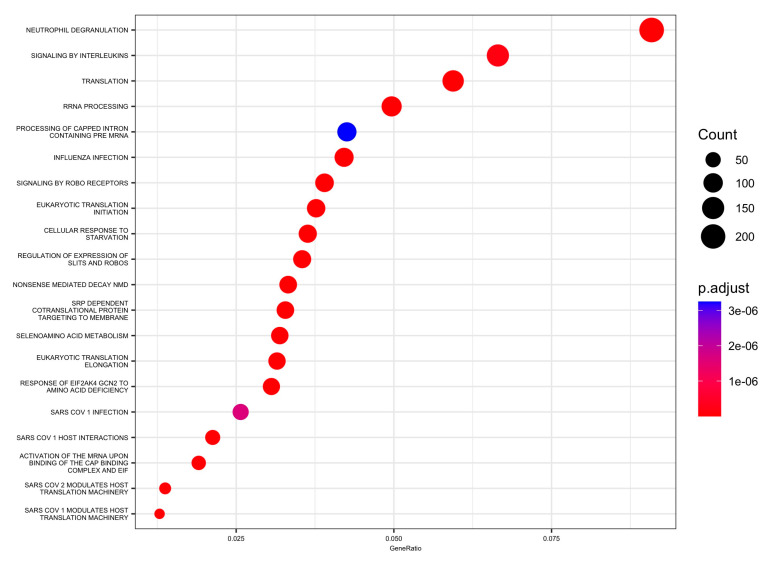
Enrichment using the Reactome collection from the MSigDB: top 20 significant pathways dot plot.

**Figure 5:**
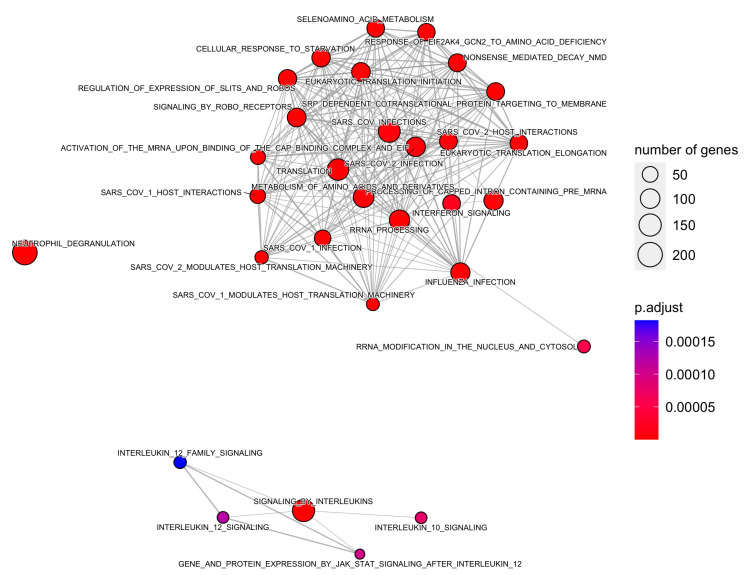
Enrichment map of *Reactome* pathways using a minimum similarity score of 0.1.

Moreover, 88 pathways in the Reactome database were identified with DEGs and small RNAs. The top enriched Reactome pathways were annotated as related to translation, neutrophil degranulation, influenza infection, rRNA processing, SRP-dependent cotranslational protein targeting to the membrane, nonsense-mediated decay (NMD), response of EIF2AK4 (GCN2) to amino acid deficiency, selenoamino acid metabolism, and others (Table 3 and Figure 4 and Figure 5).

## 4. Discussion

This is a systematic review of gene expression studies that have investigated critically ill septic patients with CAP compared to control groups (healthy adults or others). We have listed all reported DEGs in critically ill septic patients with CAP and have investigated the functional implications of these genes. The functional analyses summarize the molecular mechanisms of the inflammatory response in CAP found in those studies. Our functional analyses of DEGs were related to genes regulated by NF-kB in response to TNF, genes upregulated in response to IFN-gamma, genes defining the inflammatory response, genes upregulated by STAT5 in response to IL2 stimulation, a subgroup of regulated genes with MYC—version 1 (v1), and genes upregulated during transplant rejection.

Sepsis, a life-threatening condition, is one of the main complications of CAP. Mortality associated with CAP remains high, particularly in cases requiring ICU admission [31], even when adequate antibiotic therapy has been provided. The host immune response to sepsis can be altered in several ways, and previous studies have highlighted the complexity of this response during acute inflammation and subsequent resolution of pneumonia [32,33]. A variety of biomarkers have also been investigated, alone or in combination, for the purposes of diagnosis, etiology, mortality, and guiding antibiotic therapy, but none is ideal. Transcriptomic, proteomic, and metabolomic studies of host immune response or biomarker signatures have shown encouraging preliminary results [32].

Regarding the gene sets found in the functional analysis of the present review, studies have documented that the transcription factor MYC may be an important regulatory gene in the dysfunction of sepsis or acute respiratory distress syndrome (ARDS) secondary to sepsis [34]. On the other hand, NF-kB is an important transcriptional regulator during the process of inflammation and injury and plays a role in inflammatory disorders since it modulates the response of immunoregulatory genes, such as cytokines and chemokines, cell adhesion molecules, and antimicrobial peptides [35]. Experimental studies in animals have shown that regulation of the NF-kB signaling pathway reduces lung inflammation during pneumonia [36,37]. STAT5 signaling plays a critical role in the maintenance of lung homeostasis [38], and IFN-γ produced during pneumonia induces the transcription of target genes in the lungs, which are critical for host defenses. Studies have shown that the production of IFN-γ early during pneumonia regulates bacterial clearance [39].

In addition, 88 pathways were identified in the Reactome database. These pathways were related to translation, neutrophil degranulation, influenza infection, SARS-CoV infections, signaling by interleukins and related cytokines, IFN signaling, gene and protein expression by JAK-STAT signaling, regulation of expression of SLITs and ROBOs, immunoregulatory interactions between lymphoid and non-lymphoid cells, and others. In this regard, neutrophils are essential in the defense against invading microorganisms. Neutrophils have different tools to kill invading microbes, including distinct granule subsets that contain a variety of antimicrobial peptides and enzymes [40]. However, neutrophil granule proteins also are directly responsible for lung damage. In mice, the *Streptococcus pyogenes* M1 protein causes degranulation of all neutrophil granule subsets, leading to the accumulation of neutrophils in lung tissue, and has also been linked to increased vascular permeability and acute lung damage [41]. Moreover, cytokines are also important mediators of the innate and acquired immune response. The main functions of cytokines during an infection are cell differentiation, chemotaxis, and the regulation of inflammatory and anti-inflammatory processes. During CAP, a systemic and local inflammatory response occurs, and the number of cytokines identified and the knowledge of their functions have increased over the years. Cytokines are major players in cases of severe lung infection [42]. In addition, the Slit-induced signaling pathway is a modulator of vascular stability. Activation of this pathway reduces capillary leakage, multiorgan edema, and death in multiple animal models of infections. One experimental study found that administration of the Slit2N ligand strengthens the endothelial barrier and attenuates vascular leakage in response to the cytokine storm [43]. Furthermore, studies have suggested that HSF1 is required to initiate the host defense against bacterial infection through early activation of TLR2 signaling. HSF1(−/−) mice had a higher lung bacterial load and delayed associated inflammation compared to HSF1(+/+) mice [44].

Gene expression studies in patients with CAP have not only allowed a better understanding of the immune response but could also have applications in clinical practice. These studies have evaluated the utility of gene expression in stratifying patients into subtypes according to the prognosis of CAP, determining the fingerprints of the host response to viruses and bacteria that might be used for etiological diagnosis, and proposing new biomarkers that might be suitable for diagnosis or the development of therapies. Studies with these objectives were not included in the present systematic review. Davenport et al. [21] found that transcriptomic profiling of circulating peripheral blood leukocytes from CAP patients defined two individual sepsis response signatures (SRS1 and SRS2) related with outcomes. SRS1 was related to a higher risk of early mortality than SRS2 (14-day mortality 22% vs. 10%). The investigators documented that SRS1 was associated with an immunosuppressed phenotype. The features of this group of patients included T-cell exhaustion, endotoxin tolerance, and downregulation of human leukocyte antigen (HLA) class II. Similarly, Severino et al. [24] evaluated patterns of gene expression in blood mononuclear cells from patients with CAP and sepsis. They found that differences in oxidative phosphorylation were associated with prognosis. In addition, gene expression profiles also differed between patients who died and survived, with decreased expression of genes related to immune functions. In another study, comparison of gene expression profiles between 185 surviving and 13 non-surviving hospitalized CAP patients yielded 49 DEGs [18]. Gene set enrichment analysis found four positively enriched gene sets in survivors and seven positively enriched gene sets in the patients who died. These gen sets were related with the interferon-alpha response, apoptosis, sex hormone pathways, oxidative stress, endoplasmic reticulum stress, oxidative phosphorylation, and angiogenesis pathways.

In addition, another study reported that the whole-blood gene-expression profile of pneumonia caused by influenza A(H1N1) differed notably from those of bacterial pneumonia [45]. A total of 1416 genes were uniquely upregulated in influenza A (H1N1) pneumonia. Analysis of biological pathways revealed over-representation related to the cell cycle and its regulation, DNA damage response, apoptosis, and protein degradation. Similarly, a study evaluated gene expression using RNASeq and qPCR to discriminate bacterial from non-bacterial infectious agents in adults with lower respiratory tract infection [46]. For molecular analyses, 41 subjects were considered to have a bacterial infection, and 53 subjects were classified with a non-bacterial infection. Influenza A was the most common virus, and *Streptococcus pneumoniae* was the most common bacteria documented. The investigators found that lymphocyte, α-linoleic acid metabolism, and IGF regulation pathways including eleven genes were markers for distinguishing bacterial infection. Interestingly, Pereverzeva et al. [19] performed a study to evaluate host response biomarkers and transcriptomes between Gram-positive and Gram-negative bacteria in CAP patients in the ICU. There was no differences in blood leukocyte transcriptomes in patients with Gram-positive and Gram-negative causative pathogens.

Transcriptional expression studies have also been used to propose new biomarkers in CAP. One study has proposed the *FAIM3:PLAC8* ratio as a candidate biomarker to aid in the diagnosis of CAP in patients requiring ICU admission. The area under the curve (0.845, 95% confidence interval: 0.764–0.917) showed that the *FAIM3:PLAC8* ratio was better in discriminating between patients with CAP and without CAP compared to procalcitonin, IL8, and IL6 [27]. Likewise, an experimental study carried out in murine models of lung infection identified in its transcriptional analysis an interferon signature associated with *S. pneumoniae* infection. Likewise, the chemokines CXCL9 and CXCL10 had the best sensitivity, specificity, and predictive power to differentiate between *S. pneumoniae* and *Staphylococcus aureus* pneumonia [47]. Other investigators analyzed both protein-coding mRNA and regulatory miRNAs in peripheral blood mononuclear cells in order to identify markers that may be suitable as diagnostic biomarkers between CAP and acute exacerbations of chronic obstructive pulmonary disease (COPD) [48]. A module of 120 genes was particularly suitable to discriminate acute exacerbations of COPD and CAP and identified *HNF4A*, *MCC*, and *MUC1* as the most important discriminatory markers.

COVID-19 is a recently recognized illness, characterized as a predominantly respiratory disease that can lead to pneumonia and ARDS. Although patients with COVID-19 were excluded from the present review, several studies have evaluated gene expression profiles of this disease [49,50,51]. The gene expression profiles have been obtained from various types of samples, including whole blood; blood components, such as peripheral blood mononuclear cells, monocytes or T cells; bronchoalveolar lavage fluid; and autopsy samples. A recent review performed an analysis of the enriched pathways in COVID-19, and the main ones found were those related to regulatory aspects of the immune system. The most common pathways were associated with cytokines, especially interferons. Other pathways found were related to the immune response against bacteria, cell division and the cell cycle, organization of the extracellular matrix, exocytosis and hemostasis [49]. Moreover, a study determined the transcriptomic profile of patients with COVID-19 who required hospital admission and described those patients who developed severe disease [52]. The researchers found that a dysregulated inflammatory response was the most important factor related to severe pneumonia in SARS-CoV-2 infection. There was increased gene expression related to the pro-inflammatory state and activation of neutrophils and macrophages, in addition to a loss of immune regulation. Additionally, increased expression of genes linked to reactive oxygen species, metalloproteinases, and protein polyubiquitination was documented.

Other studies have evaluated circulating microRNAs (miRNAs) in patients with CAP or sepsis. miRNAs can regulate gene expression and are widely involved in the inflammatory response and immune regulation in infectious processes. However, miRNA research still faces several challenges, such as low sensitivity, specificity, and silencing efficiency; off-target effects; and toxic reactions [53]. Galván-Román et al. [54] assessed the usefulness of miRNA expression as a prognostic biomarker in patients with CAP who required hospital admission. High levels of miR-146a-5p and miR-16-5p were markers of good prognosis and associated with lower 30-day mortality. Likewise, another study documented that free miRNAs could be useful for diagnosis in patients with CAP or sepsis and predicting the severity of the disease [55]. miR-1246 levels were higher in patients with severe disease, while miR-193a-5p and miR-542-3p distinguished between infection (CAP or sepsis) and healthy volunteers. Interestingly, other researchers compared differential miRNA profiles between COVID-19 and CAP [56]. A signature of 15 dysregulated miRNAs was found between patients with COVID-19 and CAP. Furthermore, 4 miRNAs (miR-106b-5p, miR-221-3p, miR-25-3p, and miR-30a-5p) significantly discriminated between both diseases, with a sensitivity of 93.7% and a specificity of 89%. Regarding the 15 dysregulated miRNAs, the enriched pathways were significantly associated with angiogenesis, regulation of endothelial cells or vasodilatation, cardiac muscle cell differentiation and proliferation, leukocyte adhesion, IL6-mediated signaling, Th1 response, macrophage differentiation and MyD88-dependent Toll-like receptor signaling.

The strength of this systematic review is that only studies evaluating expression in a specific population (critical adults) and infection (CAP) were included. This means, however, that the findings are unlikely to be valid for septic patients with other etiologies or CAP patients without sepsis. The limitations of this systematic review include the exclusion of studies that were not published in English or Spanish and studies that investigated animal models or cell lines. The studies included were small and did not report sample size estimation or power analysis; the studies did not state whether statistical corrections were used for multiple testing. Some of the studies were performed in a subgroup of patients from a larger cohort but used a different methodology to determine transcriptomic analysis. For example, Khan et al. [30] assessed small RNAs, whereas Esquerdo et al. [29] studied just 35 genes using RT-PCR. However, an analysis of studies included with the cohorts using microarrays showed similar results (Figures S1 and S2).

## 5. Conclusions

This systematic review summarizes all reported DEGs in critically ill septic patients with CAP and investigates their functional implications. The results highlight the complexity of the immune response during CAP. Gene expression studies in patients with CAP not only allow a better understanding of the immune response but may also have applications in clinical practice. In this regard, future trials should evaluate the role of gene expression for diagnosis and personalized treatment approaches in CAP.

## Figures and Tables

**Table 1:** Main characteristics of the studies.

First Author, Year	Population	Control	Sample	Method	Numbers of DEGs *
Severino et al., 2014 [24]	CAP patients with sepsis	Healthy volunteers	Peripheral blood mononuclear cells	Agilent Whole Human Genome Microarray	73
Scicluna et al., 2015 [27]	CAP patients with sepsis	Healthy controls	Whole blood	Human Genome U219 96-array	1966
Esquerdo et al., 2017 [29]	CAP patients with sepsis	Healthy volunteers	Peripheral blood mononuclear cells	RT–PCR array of 35 genes involved in the NLR-inflammasome pathways	3
Burnham et al., 2017 [28]	CAP patients with sepsis	Subjects undergoing elective cardiac surgery	Peripheral blood leukocytes	Illumina HumanHT-12 v4 Expression BeadChips	1198
Khan et al., 2021 [30]	CAP patients with *S. pneumoniae* and influenza A or B	Non-infectious participants	Whole blood and plasma	Illumina TruSeq Small RNA-Seq	42 **

CAP, community-acquired pneumonia; DEGs, differentially expressed genes. * Absolute fold change (FC) > 1.7 and *p* value < 0.05. ** small RNA.

**Table 2:** Gene set enrichment Hallmark analysis of differentially expressed genes in critically ill septic patients with community-acquired pneumonia.

Gene Set Name	Gene Ratio	*p*-Value Adjusted	Brief Description
HALLMARK_MYC_TARGETS_V1	110/1190	7.68 × 10^−16^	A subgroup of genes regulated by MYC—version 1 (v1)
HALLMARK_ALLOGRAFT_REJECTION	99/1190	1.17 × 10^−10^	Genes upregulated during transplant rejection.
HALLMARK_TNFA_SIGNALING_VIA_NFKB	86/1190	0.000009	Genes regulated by NF-kB in response to TNF
HALLMARK_IL2_STAT5_SIGNALING	84/1190	0.00002	Genes upregulated by STAT5 in response to IL2 stimulation.
HALLMARK_INTERFERON_GAMMA_RESPONSE	84/1190	0.00002	Genes upregulated in response to IFNG
HALLMARK_INFLAMMATORY_RESPONSE	73/1190	0.015	Genes defining inflammatory response

NF-kB, nuclear factor kappa B; TNF, tumor necrosis factor; STAT5, signal transducer and activator of transcription 5; IL2, interleukin 2; IFN-γ, interferon-gamma.

**Table 3:** Gene set enrichment Reactome analysis of differentially expressed genes in critically ill septic patients with community-acquired pneumonia.

Gene Set Name	Gene Set Name
EUKARYOTIC_TRANSLATION_INITIATION	TRANSPORT_OF_THE_SLBP_DEPENDANT_ MATURE_MRNA
EUKARYOTIC_TRANSLATION_ELONGATION	REGULATION_OF_HSF1_MEDIATED_ HEAT_SHOCK_RESPONSE
NEUTROPHIL_DEGRANULATION	KSRP_KHSRP_BINDS_AND_DESTABILIZES_ MRNA
INFLUENZA_INFECTION	INTERFERON_GAMMA_SIGNALING
RRNA_PROCESSING	NUCLEAR_ENVELOPE_BREAKDOWN
SRP_DEPENDENT_COTRANSLATIONAL_ PROTEIN_TARGETING_TO_MEMBRANE	SUMOYLATION_OF_RNA_BINDING_PROTEINS
NONSENSE_MEDIATED_DECAY_NMD	SNRNP_ASSEMBLY
RESPONSE_OF_EIF2AK4_GCN2_TO_AMINO_ ACID_DEFICIENCY	REGULATION_OF_GLUCOKINASE_BY_ GLUCOKINASE_REGULATORY_PROTEIN
TRANSLATION	TRNA_PROCESSING
SELENOAMINO_ACID_METABOLISM	MRNA_SPLICING
CELLULAR_RESPONSE_TO_STARVATION	CELLULAR_RESPONSE_TO_HEAT_STRESS
ACTIVATION_OF_THE_MRNA_UPON_BINDING_ OF_THE_CAP_BINDING_COMPLEX_AND_EIFS_ AND_SUBSEQUENT_BINDING_TO_43S	NUCLEAR_PORE_COMPLEX_NPC_DISASSEMBLY
REGULATION_OF_EXPRESSION_OF_SLITS_ AND_ROBOS	TRNA_PROCESSING_IN_THE_NUCLEUS
SARS_COV_1_MODULATES_HOST_ TRANSLATION_MACHINERY	NUCLEOTIDE_BINDING_DOMAIN_ LEUCINE_RICH_REPEAT_CONTAINING_ RECEPTOR_NLR_SIGNALING_PATHWAYS
SIGNALING_BY_ROBO_RECEPTORS	SUMOYLATION_OF_DNA_DAMAGE_ RESPONSE_AND_REPAIR_PROTEINS
SARS_COV_1_HOST_INTERACTIONS	IMMUNOREGULATORY_INTERACTIONS_ BETWEEN_A_LYMPHOID_AND_A_NON_LYMPHOID_CELL
SARS_COV_2_MODULATES_HOST_ TRANSLATION_MACHINERY	HIV_LIFE_CYCLE
SIGNALING_BY_INTERLEUKINS	VIRAL_MESSENGER_RNA_SYNTHESIS
SARS_COV_1_INFECTION	NS1_MEDIATED_EFFECTS_ON_ HOST_PATHWAYS
PROCESSING_OF_CAPPED_INTRON_ CONTAINING_PRE_MRNA	DISEASES_OF_MITOTIC_CELL_CYCLE
METABOLISM_OF_AMINO_ACIDS_AND_ DERIVATIVES	TCR_SIGNALING
SARS_COV_2_HOST_INTERACTIONS	ONCOGENE_INDUCED_SENESCENCE
SARS_COV_INFECTIONS	PYRUVATE_METABOLISM_AND_CITRIC_ACID_TCA_CYCLE
SARS_COV_2_INFECTION	TRANSPORT_OF_MATURE_TRANSCRIPT_TO_CYTOPLASM
INTERFERON_SIGNALING	ASSOCIATION_OF_TRIC_CCT_WITH_ TARGET_PROTEINS_DURING_BIOSYNTHESIS
RRNA_MODIFICATION_IN_THE_ NUCLEUS_AND_CYTOSOL	SUMOYLATION_OF_UBIQUITINYLATION_PROTEINS
INTERLEUKIN_10_SIGNALING	FOLDING_OF_ACTIN_BY_CCT_TRIC
GENE_AND_PROTEIN_EXPRESSION_BY_ JAK_STAT_SIGNALING_AFTER_INTERLEUKIN_ 12_STIMULATION	REACTOME_HIV_INFECTION
INTERLEUKIN_12_SIGNALING	HOST_INTERACTIONS_OF_HIV_FACTORS
INTERLEUKIN_12_FAMILY_SIGNALING	GLYCOLYSIS
INTERACTIONS_OF_REV_WITH_HOST_ CELLULAR_PROTEINS	TRANSCRIPTIONAL_ACTIVITY_OF_SMAD2_ SMAD3_SMAD4_HETEROTRIMER
INTERLEUKIN_4_AND_INTERLEUKIN_13_SIGNALING	MITOCHONDRIAL_TRANSLATION
POSTMITOTIC_NUCLEAR_PORE_ COMPLEX_NPC_REFORMATION	DEADENYLATION_DEPENDENT_MRNA_DECAY
EXPORT_OF_VIRAL_ RIBONUCLEOPROTEINS_FROM_NUCLEUS	PERK_REGULATES_GENE_EXPRESSION
GENERATION_OF_SECOND_MESSENGER_ MOLECULES	SUMOYLATION_OF_CHROMATIN_ ORGANIZATION_PROTEINS
PD_1_SIGNALING	NUCLEOTIDE_SALVAGE
NUCLEAR_IMPORT_OF_REV_PROTEIN	PLATELET_ACTIVATION_SIGNALING_ AND_AGGREGATION
INTERACTIONS_OF_VPR_WITH_HOST_ CELLULAR_PROTEINS	SMAD2_SMAD3_SMAD4_HETEROTRIMER_ REGULATES_TRANSCRIPTION
SUMOYLATION_OF_DNA_REPLICATION_ PROTEINS	ABERRANT_REGULATION_OF_MITOTIC_G1_ S_TRANSITION_IN_CANCER_DUE_TO_RB1_DEFECTS
TRANSPORT_OF_MATURE_MRNAS_ DERIVED_FROM_INTRONLESS_TRANSCRIPTS	BUTYRATE_RESPONSE_FACTOR_1_BRF1_ BINDS_AND_DESTABILIZES_MRNA
ANTIVIRAL_MECHANISM_BY_IFN_ STIMULATED_GENES	TRISTETRAPROLIN_TTP_ZFP36_BINDS_AND_ DESTABILIZES_MRNA
METABOLISM_OF_NUCLEOTIDES	TRANSLATION_OF_SARS_COV_2_ STRUCTURAL_PROTEINS
SUMOYLATION_OF_SUMOYLATION_PROTEINS	CONVERSION_FROM_APC_C_CDC20_TO_ APC_C_CDH1_IN_LATE_ANAPHASE
SUMOYLATION	REGULATION_OF_MRNA_STABILITY_BY_ PROTEINS_THAT_BIND_AU_RICH_ELEMENTS

## Data Availability

Not applicable.

## Supplementary Materials

The following supporting information can be downloaded at: https://www.mdpi.com/article/10.3390/biomedicines11102755/s1. Table S1: Codes used in R to analyze the data; Table S2: Quality assessment of included studies using the modified ‘Q-Genie’ assessment tool; Table S3: Differentially expressed genes in the studies; Figure S1: Enrichment analysis using the Hallmark collection from the MSigDB: dot plot of enriched gene sets from studies included with the cohorts using microarrays; Figure S2: Enrichment using the Reactome collection from the MSigDB: top 20 significant pathways dot plot of studies included with the cohorts using microarrays.
