# Supplementary material for: A Systematic Review of Gene Expression Studies in Critically Ill Patients with Sepsis and Community-Acquired Pneumonia
Source: Biomedicines. 2023 Oct 11;11(10):2755. doi: 10.3390/biomedicines11102755 (PMC10604146; doi:10.3390/biomedicines11102755)

Supplementary Figure S1. Enrichment analysis using the Hallmark collection from the MSigDB: dot plot of enriched gene sets from studies included with the cohorts using microarrays [24,27,28].

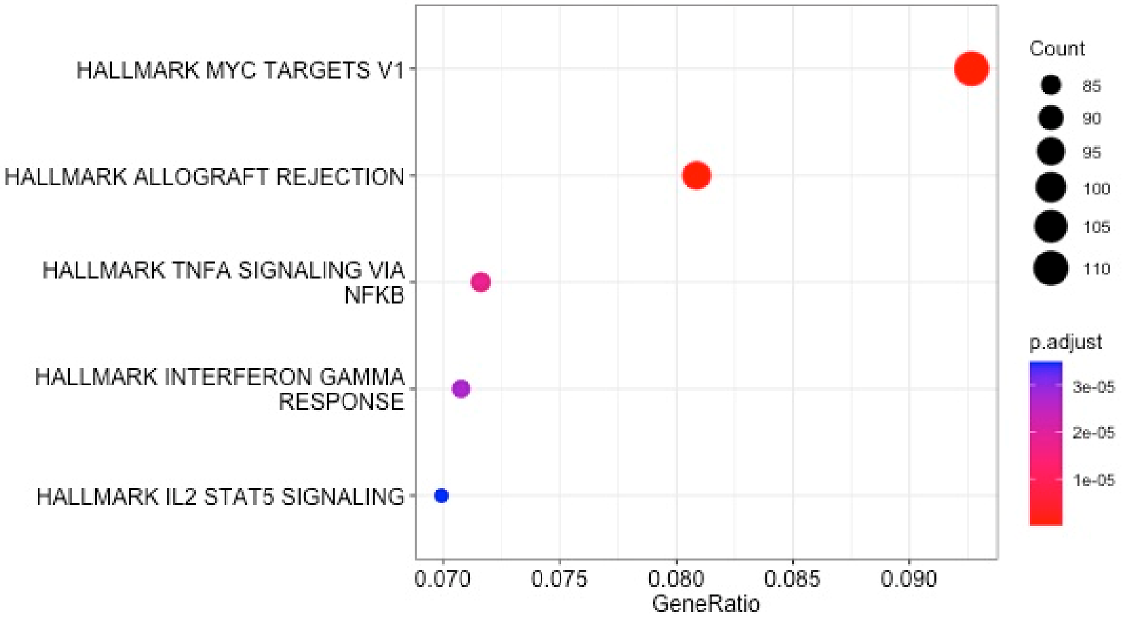

Supplement: Supplementary file 1 [file biomedicines-11-02755-s001.zip › Figure S1.pdf]
