# Supplementary material for: A Systematic Review of Gene Expression Studies in Critically Ill Patients with Sepsis and Community-Acquired Pneumonia
Source: Biomedicines. 2023 Oct 11;11(10):2755. doi: 10.3390/biomedicines11102755 (PMC10604146; doi:10.3390/biomedicines11102755)

Supplementary Figure S2. Enrichment using the Reactome collection from the MSigDB: Top 20 significative pathways dot plot of studies included with the cohorts using microarrays [24,27,28].

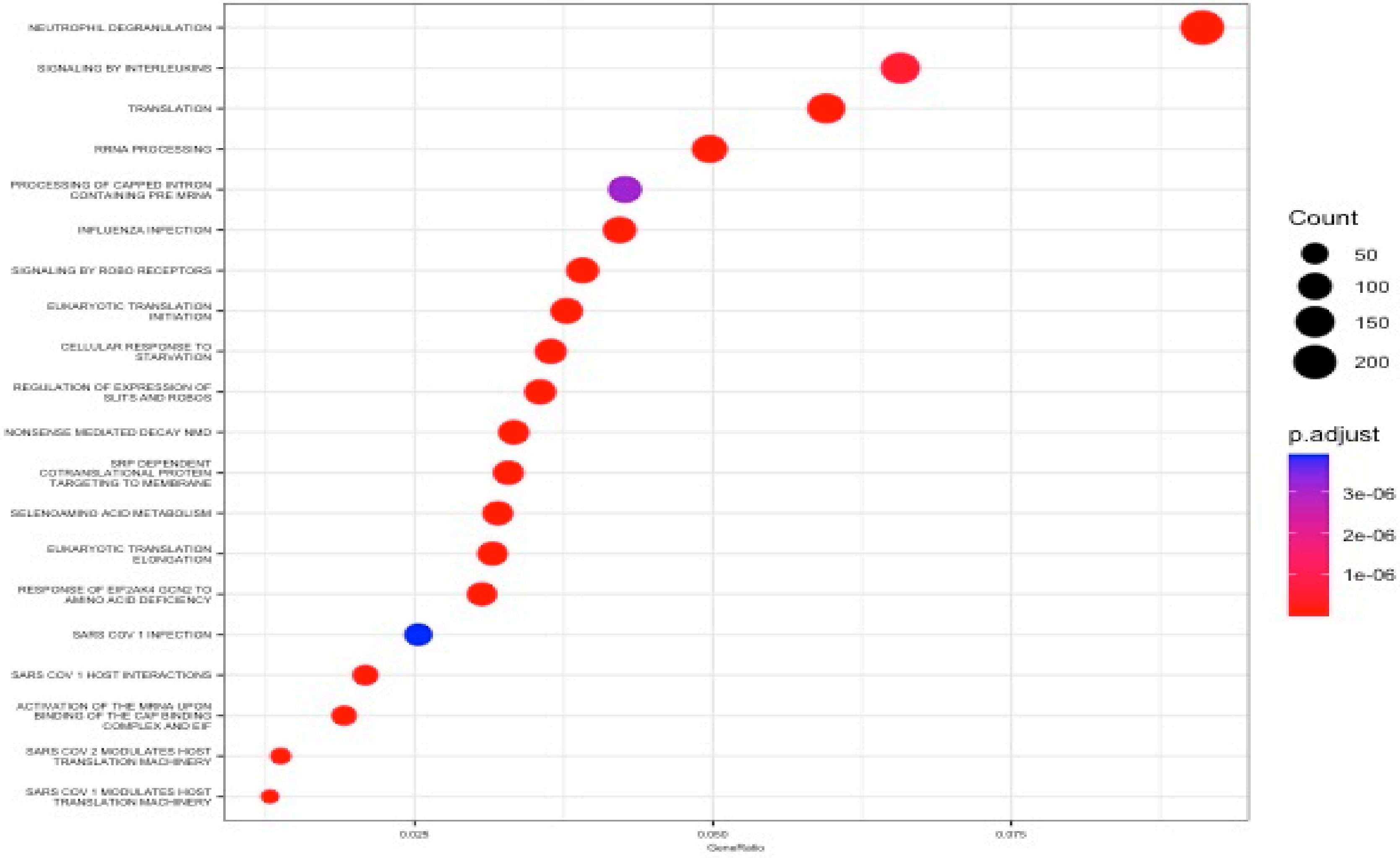

Supplement: Supplementary file 1 [file biomedicines-11-02755-s001.zip › Figure S2.pdf]
