# Supplementary material for: A Systematic Review of Gene Expression Studies in Critically Ill Patients with Sepsis and Community-Acquired Pneumonia
Source: Biomedicines. 2023 Oct 11;11(10):2755. doi: 10.3390/biomedicines11102755 (PMC10604146; doi:10.3390/biomedicines11102755)
Supplement: Supplementary file 1 [file biomedicines-11-02755-s001.zip › Table S1_RCode.pdf]

Supplementary Table S1. Codes used in R to analyze the data

```
project<-"2023_JCarrat_RSist"
wd <- getwd()
resultsDir <- file.path(wd,"results")

gmtDir <- file.path(wd,"AnalysisFiles/GSEA/gmtFiles")

# Libraries
library(gplots)
library(RColorBrewer)
library(tidyverse)
library(readxl)
library(reshape2)
library(ggpubr)
library(grid)
library(clusterProfiler)
library(enrichplot)
library(UpSetR)
library(knitr)
library(kableExtra)
library(DT)

library(AnalysisFunctions) #GitHub/MARGenomics
library(BasicPlots) #GitHub/VHIOinformatics


## burnham

bDir <- file.path(wd,"Burnham")

bTable <- read_excel(file.path(bDir,"burnham_supplementary_ table_e1.xlsx"),
           sheet = "I. CAP and FP vs control DE") #previously F. Sepsis vs Control DE

bDEG <- bTable %>% mutate(FC =  sign(`Log2 Fold Change CAP`)*2^abs(`Log2 Fold Change CAP`))
%>%
  filter(abs(FC)>1.7 & `P Value_CAP` < 0.05) %>%
  select(Gene) %>% pull()

bDEG.l <- length(unique(bDEG))
```

```
## esquerdo

eDir <- file.path(wd,"Esquerdo")
eTableAll <- read_excel(file.path(eDir,"Esquerdo.SupT1.cei12971-sup-0002-suppinfo2.xlsx"))

genes10 <-
c("CARD6","CASP5","IL10","IL18","IL1B","NLRC4","NLRP1","NLRP3","TNF","TFNAIP3")
FC10 <- c(1.7259, 1.6884, 2.451, 1.8261, 4.1022, 1.976, -1.6496, 2.375, 2.5498, 1.6389)

eTable <- data.frame(gene=genes10, FC=FC10)

eDEG <- eTable[abs(eTable$FC)>1.7, "gene"]

eDEG.l <- length(eDEG)


## khan

kDir <- file.path(wd,"Khan")
kTable <- read_excel(file.path(kDir,"Khan.SupT1.xlsx"), skip = 4)
kTable <- kTable[-1,] #first line is juist info
kTable <- kTable[!is.na(kTable$Gene),]

kDEG <- unique(kTable %>%
		mutate(FC = sign(`log2FoldChange`)*2^abs(`log2FoldChange`)) %>%
		filter(abs(`FC`)>1.7 & `pvalue` < 0.05) %>%
		select(Gene) %>% pull()) #not only miRNAs

kDEG.l <- length(unique(kDEG))



## scicluna

scDir <- file.path(wd,"Scicluna")
scTable <- read_table2(file.path(scDir,"GSE65682.top.table.CAP.HS.tsv"))

scDEG <- unique(scTable %>%
		mutate(FC = sign(`logFC`)*2^abs(`logFC`)) %>%
		filter(abs(`FC`)>1.7 & `P.Value` < 0.05) %>%
		select(Gene.Symbol) %>% pull())

scDEG.l <- length(unique(scDEG))

## Severino

seDir <- file.path(wd,"Severino")
```

```
seTable <- read_excel(file.path(seDir,"Severino.2014.Supp S1.xlsx"))

seDEG <- seTable$`Gene symbol`

seDEG.l <- length(unique(seDEG))


## common upsetr

library(UpSetR)

all <- unique(c(bDEG, eDEG, seDEG, scDEG, kDEG))

bDEG.b <- as.numeric(all %in% bDEG)
eDEG.b <- as.numeric(all %in% eDEG)
seDEG.b <- as.numeric(all %in% seDEG)
scDEG.b <- as.numeric(all %in% scDEG)
kDEG.b <- as.numeric(all %in% kDEG)

all.df <- data.frame("Burnham" = bDEG.b,
                  "Esquerdo" = eDEG.b,
                  "Severino" = seDEG.b,
                  "Scicluna" = scDEG.b,
                  "Khan" = kDEG.b,
                  row.names=all)

upset(all.df, order.by="freq")


DEGAll <- unique(c(bDEG,
                eDEG,
                seDEG,
                scDEG,
                kDEG)) #miRNAs from Khan et al. added

## ORA Hallmark

H <- read.gmt(file.path(gmtDir,"v2023/h.all.v2023.1.Hs.symbols.gmt"))

all.H <- enricher(DEGAll, TERM2GENE=H)
all.H.s <- all.H
all.H.s@result <- all.H.s@result[all.H.s@result$p.adjust<0.05,]

datatable(all.H.s@result)

dotplot(all.H.s )
all.H.s <- pairwise_termsim(all.H.s)
```

```
emapplot(all.H.s, cex_label_category = 0.4,layout="kk", cex_line=0.2, repel=T, min_edge=0.1,
max.overlaps=100)

## ORA Reactome

R <- read.gmt(file.path(gmtDir,"v2023/c2.cp.reactome.v2023.1.Hs.symbols.gmt"))

all.R <- enricher(DEGAll, TERM2GENE=R)
all.R.s <- all.R
all.R.s@result <- all.R.s@result[all.R.s@result$p.adjust<0.05,]
all.R.s@result$Description <- gsub("REACTOME_","",all.R.s@result$Description)
all.R.s@result$Description <- strtrim(all.R.s@result$Description, 70)

datatable(all.R.s@result)

dotplot(all.R.s, showCategory=20,font.size=5)
all.R.s <- pairwise_termsim(all.R.s)
emapplot(all.R.s, cex_label_category = 0.4,layout="kk", cex_line=0.2, repel=T, min_edge=0.1,
max.overlaps=100)
```
