# Supplementary material for: A Systematic Review of Gene Expression Studies in Critically Ill Patients with Sepsis and Community-Acquired Pneumonia
Source: Biomedicines. 2023 Oct 11;11(10):2755. doi: 10.3390/biomedicines11102755 (PMC10604146; doi:10.3390/biomedicines11102755)
Supplement: Supplementary file 1 [file biomedicines-11-02755-s001.zip › Table S2_Quality.pdf]

Supplementary Table S2: Quality assessment of included studies using the modified 'Q-Genie' assessment tool												
**Study**	**#1**	**#2**	**#3**	**#4**	**#5**	**#6**	**#7**	**#8**	**#9**	**#10**	**#11**	**Total score**
Severino et al., 2014 [24]	6	4	5	6	4	4	5	5	4	5	6	56
Scicluna et al., 2015 [27]	6	4	5	5	4	4	5	4	4	5	6	52
Esquerdo et al., 2017 [29]	6	5	6	6	3	3	4	6	4	5	6	54
Burnham et al., 2017 [28]	6	5	5	5	3	3	5	6	5	5	6	53
Khan et al., 2021 [30]	6	4	5	5	3	4	5	4	5	5	5	52

#1: Rationale for study; #2: Selection and definition of outcome of interest; #3: Selection and comparability of comparison group; #4: Technical classification of the exposure; #5: Non-technical classification of the exposure; #6: Other sources of bias; #7: Sample size and power; #8: A priori planning of analyses; #9: Statistical methods and control for confounding; #10: Testing of assumptions and inferences for gene expression analyses; #11: Appropriateness of inferences drawn from results.
